# Relationship between different domains of physical activity and positive mental health among young adult men

**DOI:** 10.1186/s12889-020-09175-6

**Published:** 2020-07-16

**Authors:** Kaija Appelqvist-Schmidlechner, Jani P. Vaara, Tommi Vasankari, Arja Häkkinen, Matti Mäntysaari, Heikki Kyröläinen

**Affiliations:** 1 Finnish Institute for Health and Welfare / Centre for Military Medicine, P. O Box 30, 00271 Helsinki, Finland; 2 The Department of Leadership and Military Pedagogy, National Defence University, Helsinki, Finland; 3 UKK institute for Health Promotion, Tampere, Finland; 4 Department of Physical Medicine and Rehabilitation, Health Sciences / Central Hospital of Central Finland, University of Jyväskylä, Jyväskylä, Finland; 5 Centre for Military Medicine, Helsinki, Finland; 6 Department of Biology of Physical Activity, University of Jyväskylä, Jyväskylä, Finland

**Keywords:** Physical activity, Mental health, Mental health promotion, Physical inactivity, Positive mental health, Warwick-Edinburgh mental wellbeing scale, Mental wellbeing, Leisure time physical activity, Commuting, Occupational physical activity

## Abstract

**Background:**

There is growing evidence on positive effects of physical activity (PA) on mental health. However, the focus of previous research on this relationship has typically been on mental health from the perspective of mental health problems rather than from the perspective of mental wellbeing. Further, previous research has commonly focused rather on leisure time PA without evidence on the role of other domains of PA. The aim of the present cross-sectional study was to investigate the relationship between positive mental health (PMH) and different domains of PA in young Finnish men. The secondary aim was to examine the reasons for physical inactivity among individuals with a low level of PMH.

**Methods:**

Positive mental health (measured with Short Warwick-Edinburgh Mental Wellbeing Scale, SWEMWBS), self-reported leisure time, occupational and commuting PA as well as reasons for physical inactivity were measured using questionnaires (*n* = 456, mean age 29 years) among young Finnish males. Logistic regression modelling was used to generate odds for low and high levels of positive mental health for different levels of PA and sociodemographic variables.

**Results:**

A weak positive association between leisure time PA and PMH was found in men with a low level of PMH (OR = 0.33, 95% CI 0.13–0.86). No association was found in the domains of commuting and occupational PA. Multivariate logistic regression analysis showed lower level of leisure time PA, unemployment and being single independently predicting low level of PMH. No associations were found between any domains of PA and high level of PMH. The most common reasons for physical inactivity among men with a low level of PMH were lack of interest (28%) and unwillingness to practise sports alone (27%).

**Conclusions:**

The relationship between physical activity and positive mental health seems to vary between different domains of physical activity. The findings highlight the important role of leisure time physical activity, particularly in men with a low level of positive mental health. Strategies aimed at increasing physical activity for mental health benefits should focus particularly on providing opportunities for leisure time physical activity involving social interactions for men with lower mental wellbeing.

## Background

It is widely acknowledged that the health benefits of physical activity (PA) are not limited to physical health. There is growing evidence on positive effects of PA on mental health [1–4] and on a positive association between PA and mental health [1–3, 5–14]. However, the focus of research on this relationship has more commonly been on mental health from the perspective of mental health problems or mental ill-health [2, 3, 5–9, 15] rather than from the perspective of mental wellbeing and mental health as a resource (positive mental health).

The concept of positive mental health (PMH) is based on Antonovsky’s [16] theory of salutogenesis and orientation of mental health, which focuses on health and resources for wellbeing rather than diseases providing a positive paradigm approach to the promotion of health and wellbeing. According to this paradigm, efforts and resources should be invested in mental health promotion and not just in the treatment of mental illness, for example by promoting protective factors for mental health, such as physical activity [1]. The term “positive mental health” is often used as a synonym for mental wellbeing and can be defined as “a state of wellbeing in which the individual realizes his or her own abilities, can cope with the normal stresses of life, can work productively and fruitfully, and is able to make a contribution to his or her community” [17]. Keyes [18] describes the presence of PMH as flourishing and the absence of PMH as languishing. Studies by Keyes et al. [19] have shown that high level of PMH (flourishing) can protect from suicidal ideation and problems in learning regardless of presence or absence of mental distress. Furthermore, Keyes [20] has provided evidence that the functional capacity in daily life among adults with psychiatric diagnoses and either moderate or flourishing mental health is not worse than among adults who have no mental disorders but are languishing. According to Keyes, the lack of PMH may have the same consequences on an individual’s functional capacity as the presence of illness [21]..

Physical activity (PA) – as one of the protecting factors for mental health [1] – consists of different domains, such as leisure time, commuting and occupational PA. These domains differ by their frequency, duration and intensity, and the nature of the physical activity. Commuting PA, for example, is typically characterised as a moderate aerobic activity practised either by walking or cycling to work or study, whereas leisure time PA is a structured and planned activity, which takes place in the individual’s discretionary time and is chosen on the basis of personal needs and interests. Occupational PA is an activity during the working hours at a work site, and usually includes prolonged activity including standing, walking and lifting and their combinations, sometimes with adverse circumstances (e.g. heavy loading of small muscle groups, high temperature, awkward posture). Previous studies including all these domains of PA have shown that leisure time PA is associated with mental wellbeing whereas other domains, such as commuting and occupational activity show more contradictory findings [14, 22–27]. The mechanism of effect of PA on mental health has been identified as being psychological (for example through increased self-efficacy) and/or physiological (for example through endorphins) [28].

Despite of growing evidence on a positive association between PA and PMH [22, 24, 29–32], this topic has not been adequately explored from the perspective of factors predicting low and high level of PMH. These factors are known to be partly different and to follow different patterns [24, 33–35]. Tamminen et al. [24] found in their Finnish population-based study that physical inactivity was strongly related to higher odds of low PMH, whereas no association was found between total PA and high PMH. Within the different domains of PA, only commuting PA was positively associated with high PMH, whereas in terms of low PMH, inactivity in leisure time PA and commuting PA were both related to higher odds of low PMH.

In order to develop effective public health practice, understanding of gender-specific factors contributing not only to mental health problems but also to mental wellbeing is needed. More evidence on the relationship between PMH and PA is needed in specific population groups – for example in gender groups - in order to maximize the mental health benefits of interventions aiming at promoting PA. Further, in order to encourage individuals to be physically active, it is important to understand the different preferences in PA and potential reasons for physical inactivity. As findings of previous studies indicate differences in mental wellbeing between the least and most active individuals [36] and suggest that individuals with poorer mental health are less likely to engage in PA [37, 38], knowledge on reasons for inactive lifestyle among those with a low level of PMH is needed in order to promote PA for mental health benefits. Especially for those with languishing mental health. Further, as previous studies have demonstrated gender differences in motivating and inhibiting factors for PA [39–41] as well as in mental health benefits of PA [42], gender specific evidence is needed. Gender specific initiatives for mental health promotion are needed also due to differences in mental health status and help seeking behaviour between the genders. Although mental health problems are more prevalent in women than in men, suicide rates are worldwide much higher in men compared with women ([43] WHO 2002.)

The aim of the present study was to investigate the relationship of different domains of PA with PMH among young adult men in Finland. As factors predicting high and low level of PMH are known to be partly different and associations with low level of PMH follow a different pattern than associations with high level of PMH [33], we wanted to explore PMH from both perspectives. The secondary aim was to examine the reasons for physical inactivity among individuals with a low level of PMH. Young men being a hard-to-reach group in health surveys, evidence on this topic is needed to widen our understanding of how to promote physical activity in this particularly challenging group of young men.

## Methods

The study sample consisted of young adult Finnish men who were called up to military refresher training in 2015 in Finland (convenience sample). Participation in the present study was voluntary and of 823 course participants, 792 participated in seven different measurement sites around the country. Of these, positive mental health was assessed in five measurement sites resulting in a total of 456 men (mean ± SD age 29 ± 7 years), who comprised the sample of this study.

The reservists were informed about the study in the call up letter to the military refresher training. All examinations were performed and the data were gathered at the beginning of the training. Written informed consent was received from all study participants. The ethical approval for the study was granted by the Central Finland Health Care District, and the Headquarters of the Finnish Defence Forces gave a permission to conduct the study (AM5527).

### Measures

*Positive mental health* was measured with the 7-item version of Warwick-Edinburgh Mental Wellbeing Scale (SWEMWBS) [44], widely used in population-based studies [35, 45]. Respondents are asked to rate their feelings over the previous two weeks from 1 (none of the time) to 5 (all of the time) on the following questions: “I’ve been feeling optimistic about the future”, “I’ve been feeling useful”, “I’ve been feeling relaxed”, “I’ve been dealing with problems well”, “I’ve been thinking clearly”, “I’ve been feeling close to other people” and “I’ve been able to make up my own mind about things”. Weighted sum score was calculated, higher score indicating better mental wellbeing. The permission to use the Finnish version of the scale was received from the Finnish Institute for Health and Welfare.

*Self-reported leisure time PA* was determined from responses to a single question “Which of the following definitions best describes your leisure time physical activity habits? (Think of the last 3 months and consider all leisure-time physical activity that lasted at least 20 minutes per session)” with six response categories: 1 = less than once a week, 2 = no vigorous activities, but light or moderate physical activity at least once a week, 3 = brisk physical activity once a week, 4 = vigorous activity twice a week, 5 = vigorous activity three times a week and 6 = vigorous activity at least four times a week. For further analysis, responses were binned into four categories indicating inactive (response 1), low (response 2), medium (responses 3–5) and high (response 6) leisure time PA. The leisure time PA question used in the present study has been validated against fitness, observing that vigorous PA showed a consistent dose-response relationship with cardiorespiratory and muscular fitness [46].

*Occupational PA* was measured with a single question: “Which description most accurately describes your pattern of physical activity at work?” The response alternatives were 1 = mainly sitting / office work (inactive), 2 = walking around, but no lifting or carrying heavy items (low), 3 = walking around a lot, lifting and carrying heavy items, walking upstairs (moderate) and 4 = heavy physical work (high). Good repeatability of single item occupational PA questions similar to the one used in the present study has been shown in previous studies [47, 48].

*Commuting PA* was measured with a structured question (“How much time do you spend daily on walking or cycling from home to work or other places on a regular basis?”) and classified as 1 = no daily commuting physical activity (walking or cycling), 2 = one to 29 min walking or cycling daily and 3 = ≥30 min walking or cycling daily. To the best of our knowledge, this single question has not been validated previously, although it has been widely used in earlier population-based studies (e.g. [49]).

Study participants were asked to determine *reasons for not being physically active* in their leisure time in a structured question with 15 different alternatives such as no interest for physical activity, diseases or lack of time (yes/no). The respondents were allowed to select as many options as they wanted. The options were based on selected items of the study by Aaltonen et al. [50].

### Statistical analysis

First, variance analysis / t-tests were used to determine the significance of any differences in PMH with the help of mean scores of SWEMWBS in different subgroups. Post hoc tests were performed (Bonferroni method) to investigated which subgroup differs from the rest. Then, SWEMSBS scores were classified into three categories [33]: low (scores more than one standard deviation below the mean; cores ≤21,01), moderate (cores 21,02–27,09) and high (scores more than one standard deviation above the mean; cores ≥27,10) PMH [51]. In order to better illustrate the role of contributing factors, unadjusted (model 1), partially adjusted (model 2) and fully adjusted (model 3) logistic regression modelling was used to generate odds for 1) low PMH compared with moderate and high range and for 2) high PMH compared to moderate and low range for different levels of PA and sociodemographic variables. The first model (model 1) included only the variable of PA domain (leisure-time PA, commuting PA or occupational PA) and the variable of high / low PMH. Model 2 was adjusted by age and educational level (comprehensive/secondary/ higher education) and model 3 by age, educational level (comprehensive/secondary/ higher education), marital status (married or cohabiting/single), employment status (employment or studying/unemployed or other situation), leisure time PA (inactive/low/medium/high), occupational PA (inactive/low/medium/high) and commuting PA (inactive/low/medium/high). Further, a multivariate logistic regression model was formed to generate the predictors of low and high level of PMH. For the second aim of the study, respondents with low PMH were compared with respondents with moderate and high PMH by responses given for reasons for not being physically active using Chi square -tests.

## Results

Most (80%) of the study participants reported to have been employed, 15% to have been studying and 5% to have been unemployed or not in employment or education during the last 12 months. Of the study participants, 6% had a primary, 65% a secondary and 29% a higher education. Over half of them (54%) were married or cohabiting and the rest lived alone. In terms of leisure time PA, 11% could be classified as inactive (leisure time PA less than once a week), 16% as classified for low (no vigorous activities, but light or moderate PA at least once a week), 51% for moderate (at least brisk or vigorous activity one to three times a week) and 21% for high (vigorous activity at least four times a week) leisure time PA group. One third (33%) reported to be engaged in sedentary work, 17% reported work to include low, 29% moderate and 21% high level of physical activity. One fifth (21%) reported not to walk or cycle for commuting, 49% reported to commute less than 30 min, 17% 30–59 min and 13% at least 60 min a day.

Regarding to the mean scores of SWEMWBS, PMH was associated with age, educational level, marital and employment status as well as with leisure time PA and occupational PA, but not with commuting PA (Table 1). Higher age and educational level, intimate relationship, involvement in work or education and higher level of leisure time PA predicted higher level of PMH. In terms of occupational PA, the level of PMH was the highest among men having low level of work-related PA.

**Table 1:** Mean score of SWEMWBS in different sociodemographic groups

Variable	n	Mean (SD)	p
**Age**			<.001
24 years or younger	133	23.23 (2,66)	
25–29 years	156	23.67 (2,91)	
30 years or older	157	25.05 (3,05)*** **^a^	
**Educational level**			<.001
Comprehensive school	26	23.78 (3,29)	
Secondary education	293	23.53 (2,79)	
Higher education	132	25.22 (3,22)***^a^	
**Marital status**			<.001
Married / cohabiting	246	24.78 (2,90)	
Single	206	23.18 (3,00)***	
**Employment status**			<.001
Employment / studying	361	24.30 (2,94)	
Unemployed or other situation	78	22.95 (3,13)***	
**Leisure time PA**			,015
Inactive	51	22.95 (2,65)	
Low	74	23.72 (3,91)	
Medium	231	24.21 (3,04)*	
High	96	24,51 (2,86)*	
**Occupational PA**			.042
Sedentary	189	23.99 (3,22)	
Low	66	25.02 (3,19)	
Medium	114	23.73 (2,79)* ^a^	
High	81	23.90 (2,76)	
**Commuting PA**^**a**^			ns
Not at all	92	24.17 (3,01)	
Less than 30 min daily	211	24.02 (2,96)	
30 min or more daily	126	24.18 (2,90)	

^a^Restricted to those who are employed or studying compared to the subgroup listed first in each variable: * *p* < 0.05, ** *p* < .01, *** *p* < 0.001

***^a^*p* < 0.001 Compared to the subgroup listed second in the variable

* ^a^*p* < 0.05 Compared to the subgroup listed second in the variable

According to the logistic regression models, PMH was inversely associated with leisure time PA in individuals with low PMH in unadjusted (model 1) and partially adjusted (model 2) models (Table 2). However, the association was no longer present when adjusting with marital and employment status, as well as with other domains of PA (occupational PA and commuting PA, model 3). Individuals belonging to the high occupational PA group had higher likelihood for worse PMH compared to the sedentary occupational PA group in unadjusted model only. No associations were found between any domain of PA and high level of PMH either in unadjusted or adjusted models.

**Table 2:** Odds ratios (OR) and 95% confidence intervals (CI) separately for low and high level of positive mental health (PMH) in different domains of physical activity

	Model 1^a^ OR (95% CI, n)	Model 2 ^b^ OR (95% CI, n)	Model 3 ^c^ OR (95% CI, n)
**Low level of PMH**			
**Leisure time PA**			
Inactive	1 (*n* = 51)	1 (*n* = 50)	1 (*n* = 47)
Low	0.74 (0.32–1.73, *n* = 74)	0.84 (0.35–1.98, *n* = 72)	1.27 (0.50–3.62, *n* = 67)
Medium	0.39 (0.18–0.82, *n* = 231)*	0.45 (0.21–0.95, *n* = 227)*	0.58 (0.25–1.35, *n* = 214)
High	0.30 (0.12–0.77, *n* = 96)*	0.33 (0.13–0.86, *n* = 92)*	0.38 (0.13–1.05, *n* = 90)
**Occupational PA**			
Sedentary	1 (*n* = 146)	1 (n = 146)	1 (*n* = 145)
Low	1.31 (0.54–3.17, n = 74)	1.32 (0.53–3.25, *n* = 68)	1.49 (0.58–3.86, *n* = 66)
Medium	1.94 (0.95–3.97, *n* = 129)	1.54 (0.73–3.24, *n* = 125)	1.65 (0.75–3.63, *n* = 120)
High	2.33 (1.09–4.95, *n* = 91)*	1.72 (0.78–3.78, n = 90)	1.95 (0.84–4.53, *n* = 87)
**Commuting PA**^d^			
Not at all	1 (*n* = 95)	1 (*n* = 94)	1 (n = 90)
Less than 30 min daily	0.90 (0.45–1.79, *n* = 223)	0.97 (0.48–1.94, *n* = 219)	1.13 (0.49–2.61, *n* = 207)
30 min or more daily	1.02 (0.48–2.13, *n* = 134)	1.12 (0.53–2.39, n = 129)	0.92 (0.36–2.36, *n* = 121)
**High level of PMH**			
**Leisure time PA**			
Inactive	1 (n = 51)	1 (n = 50)	1 (n = 47)
Low	1.04 (0.28–3.88, n = 74)	0.79 (0.20–3.05, n = 72)	0.61 (0.15–2.54, n = 67)
Medium	1.62 (0.54–4.84, n = 231)	1.17 (0.37–3.62, n = 227)	0.99 (0.31–3.14, n = 214)
High	1.37 (0.41–4.60, n = 96)	1.03 (0.29–3.67, n = 92)	0.93 (0.26–3.39, n = 90)
**Occupational PA**			
Inactive	1 (n = 146)	1 (n = 146)	1 (n = 145)
Low	1.11 (0.49–2.54, n = 74))	1.26 (0.52–3.07, n = 68)	1.08 (0.42–2.75, n = 66)
Medium	0.53 (0.23–1.23, n = 129)	0.88 (0.35–2.21, n = 125)	1.00 (0.39–2.58, n = 120)
High	0.78 (0.34–1.82, n = 91)	1.25 (0.46–3.36, n = 90)	1.32 (0.47–3.65, n = 87)
**Commuting PA**^d^			
Not at all	1 (n = 95)	1 (n = 94)	1 (n = 90)
Less than 30 min daily	1.03 (0.47–2.24, n = 223)	1.02 (0.45–2.32, n = 219)	0. 81 (0.37–1.75, n = 207)
30 min or more daily	0.99 (0.42–2.34, n = 134)	1.01 (0.41–2.47, *n* = 128)	0.96 (0.42–2.22, n = 121)

^a^Unadjusted model

^b^Adjusted for age and educational level,

^c^Fully adjusted model: adjusted for age, educational level, marital status, employment status and two other domains of PA (leisure time PA, occupational PA and commuting PA)

^d^Restricted to those who are employed or studying

*statistical significance *p* < 0.05

Multivariate logistic regression modelling showed unemployment and being single as well as lower level of leisure time PA independently predicting low level of PMH, whereas only older age and being in a relationship were independently predicting high level of PMH (Table 3).

**Table 3:** Results of multivariate logistic regression analysis on predictors for low and high level of positive mental health (PMH)

	B (df)	SE	Wald	Exp(B)	95% CI	p
**Low level of PMH**						
Age	.00 (1)	.02	.01	1.00	0.95–1.05	.946
Educational level	−.49 (1)	.32	2.30	.61	0.32–1.15	.153
Employment status	.83 (1)	.35	5.64	2.30	1.16–4.57	.018*
Marital status	−.86 (1)	.32	6.98	.43	0.23–0.80	.008**
Leisure time PA	−.39 (1)	.15	6.23	.68	0.50–0.92	.013*
Occupational PA	.22 (1)	.14	2.49	1.24	0.95–1.62	.116
Commuting PA	.11 (1)	.32	.12	1.12	0.59–2.11	.924
**High level of PMH**						
Age	.06 (1)	.02	6.83	1.06	1.02–1.11	.009**
Educational level	.44 (1)	.36	1.50	1.55	0.77–3.11	.235
Employment status	−.02 (1)	.59	.00	.98	0.31–3.12	.980
Marital status	.99 (1)	.41	5.74	2.69	1.20–6.05	.017*
Leisure time PA	.06 (1)	.20	.09	1.06	0.71–1.59	.774
Occupational PA	.07 (1)	.17	.18	1.07	0.77–1.49	.659
Commuting PA	−.17 (1)	.39	.19	.85	0.40–1.80	.856

Statistical significance * *p* < 0.05, ** *p* < .01

The most common reasons for physical inactivity among men with a low level of PMH were lack of interest (28%) and time (20%), unwillingness to practise sports alone (27%), being unaccustomed to physical activity (19%), other hobbies (17%) and lack of energy (17%, Table 4). In individuals with moderate or high PMH, the most common reasons for physical inactivity were lack of time (32%), other hobbies (14%), weather conditions (13%) and unwillingness to practise sports alone (13%). Lack of facilities, equipment, skills or financial resources turned out as less significant reasons for being inactive in both groups. When comparing the reasons for being inactive, individuals with a low level of PMH reported more often lack of interest (*p* < .001), energy (*p* < .034) and financial resources (*p* < .037), unwillingness to practise sports alone (*p* < .013), being unaccustomed to physical activity (*p* < .021) and unaesthetic environment (*p* < .041) as reasons compared to individuals with higher level of PMH (Table 4).

**Table 4:** Distribution of reasons for physical inactivity among respondents with a low level of positive mental health (PMH) compared with those with a high or moderate level of PMH (%)

Reasons for physical inactivity	Low level of PMH %	Moderate or high level of PMH %	p
Health reasons	0	2	.370
Lack of interest	28	8	<.001***
Weather conditions	13	13	1.000
Unaesthetic environment	9	3	.041*
Being unaccustomed to physically active	19	8	.021*
Other hobbies	17	14	.569
Lack of energy	17	8	.034*
Lack of time	20	32	.077
Sports facilities are too far away	5	3	.713
Lack of skills needed for sports	5	2	.161
Lack of equipment needed for sports	3	2	.322
Fear of getting hurt during sports	2	1	.372
Lack of financial resources	8	2	.037*
Unwilling to practise sports alone	27	13	.013*
No reasons for being physically inactive	31	41	.167

Statistical significance * *p* < 0.05, ** *p* < .01, *** *p* < 0.001

## Discussion

The aim of the present study was to investigate the association of different domains of PA with PMH among young adult men in Finland. The study found PMH associating only in the leisure time PA domain, but no association in the domains of commuting and occupational PA was found when adjusting for confounding factors. This result is partly consistent with previous studies [9, 14, 22–24, 31, 32], even though evidence focusing specifically on PMH in young males is scarce. The meta-analysis by White et al. [27] on the relationship between different domains in physical activity and mental wellbeing showed that in adults both leisure time PA and commuting PA had a positive association with mental health, whereas work-related PA was positively associated with mental ill-health. Further, in line with the present study findings, White et al. [14] found leisure time PA but not commuting PA associating positively with affective wellbeing in adolescents. In the study of Cerin et al. [23], only leisure time PA was independently associated with mental wellbeing in a sample of Australian adults, other forms of PA being either unrelated or negatively related with mental wellbeing. They found no association with commuting PA and mental wellbeing and a negative association between occupational PA and mental wellbeing in younger adults. Ohta et al. [26] investigated the association between leisure time PA and commuting PA with mental health in a Japanese sample, indicating a positive association with mental health in both domains of PA, but only in males. Tamminen et al. [24] found PMH positively associating with leisure time PA and commuting PA, but not with occupational PA. Further, the findings also indicated that physical inactivity was strongly associated with low PMH.

The predominant role of leisure time PA in the relationship between PA and PMH could be explained by at least two possible reasons. Firstly, in terms of leisure time PA, the activity is based on own preferences and schedules which can therefore be seen more easily affecting mental wellbeing. Leisure time PA has better opportunities to provide, for example, vicissitudes, improvement in sense of self-efficacy and mastery as well as opportunities for social interactions, which are suggested to act as mechanisms between physical activity and mental health [52]. In addition, the quality of the PA experience may be essential to the mental health outcomes obtained and therefore, participating in enjoyable PA can be expected to increase the likelihood of receiving mental health benefits alongside physical benefits [13]. Secondly, in terms of both commuting and occupational PA, physical activity may be perceived as necessary rather than voluntary, for example, among individuals with lower financial resources who are active in commuting due to financial reasons. The same kind of necessity and lack of self-determination may be the issue in individuals working with tasks involving high physical demands, where the work is externally controlled.

According to the mean scores of SWEMWVS, there seemed to be a positive dose-response -relationship between the level of leisure time PA and PMH. Previous studies [6, 53, 54] have found non-linear associations between mental health and PA, but the perspective in these studies has often been mental health problems instead of mental wellbeing. One of the largest cross-sectional studies [6], based on 1.2 million individuals, suggested that regular exercise associates with better mental health, while PA more than 23 times a month or for sessions longer than 90 min is associated with worse mental health. Similar findings were found in the study of Mummery et al. [54] with a cross-sectional study design and in the longitudinal study by Dore et al. [31] suggesting that increased levels of leisure time PA do not reliably result in increased mental health status despite the positive association between PA and mental health. In these studies, however, the samples included both males and females with wide-ranging age profiles and they are therefore not fully comparable with the present study. Regarding occupational PA, there was a U-shaped curve in the dose-response relationship, showing the highest PMH values in the low occupational PA group. Interestingly, this finding is similar to what has been observed in the relationship between occupational PA and cardiovascular health [55].

This study focused more closely in two dimensions of PMH: low (languishing) and high (flourishing) levels of PMH. The results showed that the association between leisure time PA and PMH was significant primarily in the dimension of languishing mental health when adjusting with age and educational level. Thus, low level of PMH was associated with physical inactivity in leisure time, whereas there was no association between high level of PMH and leisure time PA. In the fully adjusted model (adjusted with age, educational level, marital status, employment status and other domains of PA), statistically significant relationships were no longer present. The study showed that, besides leisure time PA, there are many other variables predicting PMH, especially high PMH, such as older age and being in a relationship. This finding is supported for instance by the findings of the Scottish Health survey [56] measuring mental wellbeing with the same scale used in this study and indicating these sociodemographic background variables to be strongly associated with PMH. The sample of the present study consisted of young men with a relatively small age variation. However, young adulthood is a major transition period and the most challenges in starting independent life are commonly faced especially during the first years. This may negatively affect the mental well-being especially in the early stage of young adulthood.

As a cross-sectional study, no suggestions for causal effects or direction of causality between PMH and PA can be made. Increased leisure time PA may promote mental wellbeing and/or increased mental wellbeing may result in increased PA. Choi et al. [7] found a bidirectional relationship between PA and mental ill-health, suggesting a causal relationship between physical activity and reduced risk for depression. Meta-analyses on prospective cohort studies by Schuch et al. [3, 15] showed that PA could confer protection against the emergence of depression and anxiety regardless of demographic factors. However, also these findings are based on data from both genders with wide-ranging age profiles and do not therefore provide data specifically in younger males.

Important implications for practice were discovered by this study in terms of reasons for physical inactivity among young men with low PMH, as previous research has mainly focused on barriers for physical activity among individuals with severe mental health problems [50]. The group of men with low PMH is an important group for programme developers and policy makers, as languishing mental health has been seen as a risk for future mental health problems [33]. Among individuals with severe mental health problems, low mood and stress have been identified as the most prevalent barriers towards physical activity, followed by lack of support [57]. In the present study, besides lack of interest, time and energy, unwillingness to practise sports alone was highlighted as one significant reason for physical inactivity in this target group. This finding emphasizes the importance of providing young men with leisure time PA opportunities in different social contexts that can facilitate socializing with others. Dore et al. [31] examined the associations between the context in which PA was undertaken (team sports, informal groups, individual physical activity) and PMH in young adults. They found that particularly the context of team sports provides the benefits of PA on PMH. Therefore, providing possibilities for informal sports groups and / or team sport settings, rather than focusing only on increasing PA in general could be an effective strategy to promote mental health and to prevent depression among young men. This could benefit, especially, those physically inactive young men who have low level of PMH. The study of Harris [36] showed – besides a positive correlation between increased PA and increased mental wellbeing – that increases in mental wellbeing were significantly greater in the group of most inactive participants.

### Strengths and limitations of the study

The present study provides novel and valuable evidence on the relationship between mental wellbeing and different domains of PA in young adult males, a hard-to-reach group in health surveys. As for example suicide rates are known to be higher in men than in women [43], gender specific knowledge of different strategies to promote mental health especially in men is needed. However, there are some sampling bias due to the study setting. The data were collected in five different sites around the country, geographically representing the Finnish population in this age group quite well. However, as the sample consisted of reservists participating in a military refresher training, some biases have to be taken into account. In Finland, about 30% of every male cohort do not complete their military service due to several reasons (such as civil service or health-related problems) and thus, do not receive an invitation to the military refresher course. Consequently, the sample used in this study does not include, for instance, men with somatic or mental health problems which have prevented them to complete their military service. Men exempted from the service are known to have poorer socio-economic and mental health status [58]. Thus, the sample used in this study can be estimated to consist of young men with better health and psychosocial wellbeing status compared to a population-based sample. The impact of the site was tested by including the site in the regression models. However, no impact of the site was found in adjusted models.

Both PMH and PA were based on self-reports. PMH was measured with a well-established and validated scale and PA with series of questions commonly used in previous health surveys. In terms of PA, self-reported data can be seen as a limitation. It is known that self-reports of physical activity commonly overestimate the actual activity level [7, 59]. Further, options in the question of barriers for physical activity were selected from a previous study and, therefore, not validated before. The cross-sectional nature of the study provides no causal directions for the relationship between physical activity and positive mental health, which is discussed earlier in this paper.

## Conclusion

The study showed a weak but statistically significant positive association between leisure time PA and PMH in young men. Association was found only in individuals with a lower level of PMH, whereas such an association was not evident in individuals with high PMH. The relationship between leisure time PA and PMH seems to be complex with many affecting and confounding factors, such as employment and marital status. No association was found between PMH and occupational or commuting PA. The findings of this study emphasize the important role of leisure time PA particularly in individuals with low level PMH. In line with the conclusions of the meta-analysis by White et al. [27], the present study indicates that promoting particularly leisure time PA, compared to other domains of PA, may provide the most beneficial mental health outcomes in mental health promotion and prevention of mental ill-health in young males. Knowing that, for example, suicide rates are significantly higher in young men compared with young women [43] and that high level of PMH can protect from suicidal ideation [19], leisure time PA may provide possibilities for mental health promotion and suicide prevention strategies. Besides lack of interest, time and energy, unwillingness to practise sports alone was highlighted as one significant reason for physical inactivity in this target group. Strategies aimed at increasing PA for mental health benefits should focus particularly on providing opportunities for physical activities in social context for individuals with lower mental wellbeing. More research using longitudinal research designs, but especially RCT studies using both quantitative and qualitative methods is needed to detect the potential mechanisms behind the observed relationships. Future studies with qualitative methods are needed to widen our understanding of reasons for inactive life style in young adult males with lower mental wellbeing. One interesting question is the role of social interactions in PA, for instance, whether PA with others is more beneficial than PA alone in terms of mental health outcomes.

## Data Availability

The datasets generated and analysed during the current study are not publicly available but can be requested from the corresponding author with permission the Headquarters of the Finnish Defence Forces and University of Jyväskylä on reasonable request.

## Abbreviations

CI: Confidence interval
CPA: Commuting physical activity
OPA: Occupational physical activity
OR: Odds ratio
PA: Physical activity
PMH: Positive mental health
SWEMWBS: Short Warwick-Edinburgh Mental Wellbeing Scale

## References

[1] Biddle S (2016). Physical activity and mental health: evidence is growing. World Psychiatry.

[2] Mammen G, Faulkner G (2013). Physical activity and prevention of depression. A systematic review of prospective studies. Am J Prev Med.

[3] Schuch F, Vancampfort D, Firth J, Rosenbaum S, Ward P, Silva E (2018). Physical activity and incident depression: a meta-analysis of prospective cohort studies. Am J Psychiatry.

[4] Stubbs B, Vancampfort D, Rosenbaum S, Firth J, Cosco T, Veronese N (2017). An examination of the anxiolytic effects of exercise or people with anxiety and stress-related disorders: a meta-analysis. Psychiatry Res.

[5] Bennie J, Teychenne M, De Cocker K, Biddle S (2019). Associations between aerobic and muscle-strengthening exercise with depressive symptom severity among 17,839 U-S adults. Prev Med.

[6] Chekroud S, Gueorguieva R, Zheutlin A, Paulus M, Krumholz H, Krystal J (2018). Association between physical exercise and mental health in 1.2 million individuals in the USA between 2011 and 2015: a cross-sectional study. Lancet Psychiatry.

[7] Choi K, Chen C, Sein M, Klimentidis Y, Wang M, Koenen K (2019). Assessment of bidirectional relationships between physical activity and depression among adults: a 2-sample Mendelian randomization study. JAMA Psychiatry.

[8] Dogra S, MacIntosh L, O’Neil C, D’Silva C, Shearer H, Smith K (2018). The association of physical activity with depression and stress among post-secondary school students: a systematic review. Ment Health Phys Act.

[9] Kettunen O, Kyröläinen H, Santila M, Vasankari T (2014). Physical fitness and volume of leisure time physical activity relate with low stress and high mental resources in young men. J Sports Med Phys Fitness.

[10] McDowell CP, Dishman RK, Gordon BR, Herring MP (2019). Physical activity and anxiety: a systematic review and meta-analysis of prospective cohort studies. Am J Prev Med.

[11] Schuch F, Stubbs B (2019). The role of exercise in preventing and treating depression. Curr Sport Med Rep.

[12] Schuch F, Vancampfort D, Richards J, Rosenbaum S, Ward P, Stubbs B (2016). Exercise as a treatment for depression: a meta-analysis adjusting for publication bias. J Psychiatr Res.

[13] Teychenne M, White R, Richards J, Schuch FB, Rosenbaum S, Bennie JA (2020). Do we need physical activity guidelines for mental health: What does the evidence tell us?. Ment Health Phys Act.

[14] White R, Parker P, Lubans D, MacMillan F, Olson R, Astell-Burt T (2018). Domain-specific physical activity and affective wellbeing among adolescents: an observational study of the moderating roles of autonomous and controlled motivation. Int J Beh Nutr Phys Act.

[15] Schuch F, Stubbs B, Meyer J, Heissel A, Zech P, Vancampfort D, et al. Physical activity protects from incident anxiety: a meta-analysis of prospective cohort studies. Depress Anxiety. 2019. 10.1002/da.22915. 10.1002/da.22915 31209958

[16] Antonovsky A (1996). The salutogenic model as a theory to guide health promotion. Health Prom Int.

[17] World Health Organization. Mental health: strengthening our response. Fact sheet; 2018. Retrieved from https://www.who.int/en/news-room/fact-sheets/detail/mental-health-strengthening-our-response.

[18] Keyes C (2002). The mental health continuum: from languishing to flourishing in life. J Health Soc Beh.

[19] Keyes C, Eisenberg D, Perry G, Dube S, Kroenke K, Dhingra S (2012). The relationship of level of positive mental health with current mental disorders in predicting suicidal behavior and academic impairment in college students. J Am College Health.

[20] Keyes C (2004). The nexus of cardiovascular disease and depression revisited: the complete mental health perspective and the moderating role of age and gender. Aging Ment Health.

[21] Keyes C (2005). Mental illness and/or mental health? Investigating axioms of the complete state model of health. J Consult Clin Psychol.

[22] Mason P, Curl A, Kearns A (2016). Domains and levels of physical activity are linked to adult mental health and well-being in deprived neighbourhoods: a cross-sectional study. Ment Health Phys Act.

[23] Cerin E, Leslie E, Sugiyama T, Owen N (2019). Associations of multiple physical activity domains with mental well-being. Ment Health Phys Act.

[24] Tamminen N, Reinikainen J, Appelqvist-Schmidlechner K, Borodulin K, Mäki-Opas T, Solin P. Associations of physical activity with positive mental health: a population-based study. Ment Health Phys Act. 2020;18. 10.1016/j.mhpa.2020.100319.

[25] Humphreys DK, Goodman A, Ogilvie D (2013). Associations between active commuting and physical and mental wellbeing. Prev Med.

[26] Ohta M, Mizoue T, Mishima N, Ikeda M (2007). Effects of the physical activities in leisure time and commuting to work on mental health. J Occup Health.

[27] White R, Babic M, Parker P, Lubans D, Astell-Burt T, Lonsdale C (2017). Domain-specific physical activity and mental health: a meta-analysis. Am J Prev Med.

[28] Peluso MAM, Guerra De Andrade LHS. Physical activity and mental health: the association between exercise and mood. Clinics. 2005;60(1):61–70.. 10.1590/s1807-59322005000100012 15838583

[29] Bakir Y, Kangalgil M (2017). The effects of sport on the level of positivity and well-being in adolescents engaged in sport regularly. J Educ Train Stud.

[30] Shaikh S, Ansari M, Sheeraz B, Kalhoro Z (2016). Quality of life and mental health among university students: a comparison of sports participants and non-participants. Res J Phys Educ Sports Sci.

[31] Dore I, O’Loughlin J, Schnitzer M, Dattta G, Fournier L (2018). The longitudinal association between the context of physical activity and mental health in early adulthood. Ment Health Phys Act.

[32] Black S, Cooper R, Martin K, Brage S, Kuh D, Stattford M (2015). Physical activity and mental well-being in a cohort aged 60-64 years. Am J Prev Med.

[33] Stewart-Brown S, Samaraweera PC, Taggart F, Kandala NB, Stranges S (2015). Socioeconomic gradients and mental health: implications for public health. Br J Psychiatry.

[34] Stranges S, Samaraweera PC, Taggart F, Kandala NB, Stewart-Brown S (2014). Major health-related behaviours and mental well-being in the general population: the health survey for England. BMJ Open.

[35] Ng Fat L, Mindell J, Boniface S, Stewart-Brown S (2016). Evaluating and establishing national norms for the short Warwick-Edinburgh mental well-being scale (SWEMWBS) using the health survey for England. Qual Life Res.

[36] Harris MA (2018). The relationship between physical inactivity and mental wellbeing: findings from a gamification-based community-wide physical activity intervention. Health Psychol Open.

[37] Stubbs B, Firth J, Berry A, Schuch FB, Rosenbaum S, Gaughran F, Veronesse N, Williams J, Craig T, Yung AR, Vancampfort D. How much physical activity do people with schizophrenia engage in? A systematic review, comparative meta-analysis and meta-regression. Schizophr Res. 2016: Published online 31 May 2016. 10.1016/j.schres.2016.05.017. 10.1016/j.schres.2016.05.017 27261419

[38] Wheeler AJ, McKenna B, Madell D (2013). Stereotypes do not always apply: findings from a survey of the health behaviours of mental health consumers compared with the general population in New Zealand. NZ Med J.

[39] Van Uffelen JGZ, Khan A, Burton NW. Gender differences in physical activity motivators and context preferences: a population-based study in people in their sixties. BMC Public Health. 2017;17:624. 10.1186/s12889-017-4540-0 PMC5496591 28676081

[40] Phongsavan P, McLean G, Bauman A (2007). Gender differences in influences of perceived environmental and psychosocial correlates on recommended level of physical activity among new Zealanders. Psychol Sport Exerc.

[41] DeWolfe CEJ, Watt MC, Romero-Sanchiz P, Stewart SH. Gender differences in physical activity are partially explained by anxiety sensitivity in post-secondary students. J Am College Health. 2020;68(3):1–4. 10.1080/07448481.2018.1549048 30645185

[42] Asztalos M, De Bourdeaudhuij I, Gardon G (2010). The relationship between physical activity and mental health varies across activity intensity levels and dimensions of mental health among women and men. Public Health Nutr.

[43] World Health Organization (2002). Gender and Mental Health.

[44] Tennant R, Hiller L, Fishwick R, Platt S, Joseph S, Weich S (2007). The Warwick-Edinburgh mental well-being scale (WEMWBS): development and UK validation. Health Qual Life Outcomes.

[45] Stewart-Brown S, Tennant A, Tennant R, Platt S, Parkinson J, Weich S. Internal construct validity of the Warwick-Edinburgh Mental Well-Being-Scale (WEMWBS): a Rasch analysis using data from the Scottish Health Education Population Survey. Health Qual Life Outcomes. 2009;7(15). 10.1186/1477-7525-7-15. 10.1186/1477-7525-7-15 PMC2669062 19228398

[46] Fogelholm M, Malmberg J, Suni J, Santtila M, Kyröläinen H, Mäntysaari M (2006). International physical activity questionnaire: validity against fitness. Med Sci Sports Exerc.

[47] Kurtze N, Rangul V, Hustvedt BE, Flanders WD (2007). Reliability and validity of self-reported physical activity in the Nord-Trøndelag health study (HUNT 2). Eur J Epidemiol.

[48] Evenson KR, McGinn AP (2005). Test-retest reliability of adult surveillance measures for physical activity and inactivity. Am J Prev Med.

[49] Barengo NC, Kastarinen M, Lakka T, Nissinen A, Tuomilehto J (2006). Different forms of physical activity and cardiovascular risk factors among 24-64-year-old men and women in Finland. Eur J Cardiov Prev Reh.

[50] Aaltonen S, Leskinen T, Alen M, Kaprio J, Liukkonen J, Kujala UM (2012). Motives for and barriers to physical activity in twin Paris discordant for leisure time physicl activity for 30 years. Int J Sports Med.

[51] Taggart F, Stewart-Brown S, Parkinson J (2016). Warwick-Edinburgh Mental Well-Being Scale (WEMWBS). User Guide (Version 2).

[52] Paluska S, Schwenk T (2000). Physical activity and mental health: current concepts. Sports Med.

[53] Bernard P, Dore I, Romain A, Hains-Monfette G, Kingsbury C, Sabiston. Dose response association of objective physical activity with mental health in a representative national sample of adults: A cross-sectional study. PLoS One. 2018;13(10):e0204682. 10.1371/journal.pone.0204682. 10.1371/journal.pone.0204682 PMC6200189 30356252

[54] Mummery K, Schofield G, Caperchione C (2004). Physical activity: physical activity dose-response effects on mental health status in older adults. Austr NZ Publ Heal.

[55] Holtermann A, Krause N, van der Beek AJ, Straker L (2018). The physical activity paradox: six reasons why occupational physical activity (OPA) does not confer the cardiovascular health benefits that leisure time physical activity does. Br J Sports Med.

[56] Wilson M, Kellock C, Adams D, Landsberg J. The Scottish health survey. Topic Report. Mental health and Wellbeing. Official statistics publication for Scotland: The Scottish Government; 2015. http://www.gov.scot/Resource/0046/00469088.pdf.

[57] Firth J, Rosenbaum S, Stubbs B, Gorczynski P, Yung AR, Vancamfort D. Motivating factors and barriers towards exercise in severe mental illness: a systematic review and meta-analysis. Psychol Med. 46(14)):2869–81. 10.1017/S0033291716001732. 10.1017/S0033291716001732 PMC5080671 27502153

[58] Appelqvist-Schmidlechner K, Upanne M, Henriksson M, Parkkola K, Stengård E (2010). Young men exempted from compulsory military or civil service in Finland – a group of men in need of psycho-social support. Scand J Public Health.

[59] Craig R, Mindell J, Hirani V (2009). Editors. Health survey for England 2008: volume 1 - physical activity and fitness. National Centre for social research and UCL medical school.

